# Tied Infections: How Social Connectedness to Other COVID-19 Patients Influences Illness Severity

**DOI:** 10.1177/00027642211003138

**Published:** 2021-12

**Authors:** Xuewen Yan, Tianyao Qu, Nathan Sperber, Jinyuan Lu, Mengzhen Fan, Benjamin Cornwell

**Affiliations:** 1 Cornell University, Ithaca, NY, USA; 2 Fudan University, Shanghai, China; 3 Shanghai Jiao Tong University, Shanghai, China; 4 University of Oxford, Oxford, UK

**Keywords:** COVID-19, social networks, illness severity, family, survival analysis

## Abstract

Expanding on recent research on the transmission of COVID-19 via social networks, this article argues that exposure to familial and other close contacts who already have the disease may increase the severity of one’s subsequent illness. We hypothesize that having family members or close contacts who were diagnosed with COVID-19 before one’s own diagnosis exacerbates illness severity due to several potential mechanisms including changes in available social support access, increased stress and strain, and increased viral load due to the nature of one’s exposure to the novel coronavirus. We analyze administrative data of all 417 patients who were diagnosed with COVID-19 in the Chinese city of Shenzhen between January 8 and February 25, 2020. Our analyses show that, when patients had family members or close ties diagnosed with COVID-19, they experienced more severe illness. We also find that patients with infected family members or close contacts did not have significantly extended total illness duration, due to their reduced time to diagnosis. The implications of both findings are discussed.

## Introduction

In the world’s ongoing battle against the COVID-19 public health crisis, family transmission has emerged as a significant source of viral exposure. Early in the outbreak when Wuhan, Hubei, was the epicenter of the disease, infections among familial clusters in China and Singapore provided evidence for researchers to confirm person-to-person spread, symptomatic or asymptomatic (Pung et al., 2020; Qian et al., 2020; Yu et al., 2020). As virus hotspots began to appear across the globe, a prevalent pattern in the spread of the virus was the importance of the link between spouses and other family members (Edwards, 2020; Sperling, 2020; Wagner, 2020). Indeed, the WHO-China Joint Mission noted that family clusters acted as a primary driver of outbreaks in non-Hubei provinces of China (World Health Organization, 2020).

The emphasis on family members and close contacts as a key locus for virus transmission has informed governments’ responses to the COVID-19 pandemic (S. Chen et al., 2020). An equally fruitful question, we argue, is whether, apart from channeling transmission, the configuration of familial or other close social ties affects COVID-19 *severity* among existing patients. This is a crucial question to consider, for both pragmatic and theoretical reasons. In order to design more effective clinical interventions, researchers need to identify various potential factors associated with COVID-19 symptom severity. This question is also important from a theoretical standpoint. Research in the sociology of health, social epidemiology, and related fields has established the pervasive influence of social networks on individuals’ health outcomes (House, Landis, et al., 1988; House, Umberson, et al., 1988; Morris, 2004; Smith & Christakis, 2008; Thoits, 2011; Zhang & Centola, 2019). These related areas of research demonstrate that the onset, severity, treatment, and resolution of individuals’ health problems are influenced by their social network members through a variety of mechanisms (e.g., health-related behaviors).

Expanding on this work, we assess whether and how being connected to a family member or other close contact who has COVID-19 impacts patients’ subsequent illness severity. We derive two complementary hypotheses that may simultaneously account for severity-related patient outcomes, drawing primarily on network theories of health but also referencing the unique variables likely operative in the pandemic context. To test our hypotheses, we analyze administrative COVID-19 patient data from a major Chinese city that covers all confirmed cases between January 8 and February 25, 2020. Our analysis involves a combination of social network visualization and survival analysis, as well as sensitivity analyses.

## Social Network Ties and Illness Severity

There is abundant evidence that rates of SARS-CoV-2 infection and COVID-19-associated mortality are disproportionately greater among members of socially disadvantaged groups—especially among nursing home residents, racial/ethnic minorities and people who have preexisting medical conditions, such as diabetes (Chowkwanyun & Reed, 2020; Esai Selvan, 2020; Jordan et al., 2020; Kim & Bostwick, 2020; Laster Pirtle, 2020; Tai et al., 2020). We expand the sociological dialogue around these conditions by arguing that the severity of the resulting COVID-19 disease also has a *relational* basis, rooted in the nature of the relationships its victims have with each other. Specifically, we argue that there are several mechanisms by which a person’s social connection to another infected individual can modulate the severity of one’s COVID-19 illness. In the following sections, we explain our rationale by examining some of these potential mechanisms.

### Social Support Change

A foundational insight from research on social networks and health is that social connections serve as an essential source of support for individuals’ psychological and physical well-being (Cohen & Syme, 1985; House, Umberson, et al., 1988; Thoits, 2011; Zhang & Centola, 2019). A robust literature has suggested that being socially connected to others protects against a variety of morbidities and all-cause mortality (Holt-Lunstad et al., 2010; Shor & Roelfs, 2015). The pathways through which social ties affect health are multifaceted, ranging from indirect psychological mechanisms such as the creation of a sense of mattering or belonging, companionship and self-esteem, to direct network influences on health behavior such as social influence, social comparison, and social control (Thoits, 2011).

Many studies have pointed to the critical role of close social ties and familial relationships in providing instrumental and socioemotional support (House, Umberson, et al., 1988; Umberson & Thomeer, 2020). For one, especially during times of illness or crisis, close family and friends are often the most reliable and resourceful contacts for an individual. They offer comfort and emotional support, help arrange for medical care, and assist with household chores and child care (Berkman & Glass, 2000). For example, at the onset of an illness episode, one’s connections to close ties tend to increase in both quantity and frequency of contact, in part reflecting their elevated need for functional support in the face of disruptive health events (Perry & Pescosolido, 2012).

An important implication is that the illness or morbidity of a close social contact can render individuals more susceptible to poor health outcomes due to the deprivation of social support. In the case of COVID-19 symptom severity, it is possible that these negative effects are amplified. Intrafamilial infection implies the simultaneous illness of the individual *and* their close tie, and thus the loss of avenue for instrumental and expressive support on both sides. Specifically, as one family member begins to show symptoms of COVID-19, another family may start serving the role of a primary caregiver by providing informal health care and taking up more household chores. In situations of intrafamilial infection, the family caregiver may also have contracted the virus. On one hand, apart from lacking access to care and support for themselves, the infected caregiver may experience enhanced caregiver burden (Dunkin & Anderson-Hanley, 1998), underplay the symptoms they experience to fulfill their caregiving role, or prioritize the health of the care recipient over their own—all of which can lead to the more rapid progression of illness. On the other hand, the illness severity of the care recipient may be directly aggravated once the caregiving family member falls ill, as the health of care recipients tends to heavily depend on the health of the caregiver due to the support and assistance the latter provides for the former (Dunkin & Anderson-Hanley, 1998; Schulz & Beach, 1999).

Another factor to consider in the social support dynamics of COVID-19 intrafamilial infection is resource limitations within the family. This is a concern that is immediately relevant to families living in the same household, although it could also apply to family members living apart who regularly provide care for each other (e.g., divorced parents who share custody of children, adult couples who live near their senior parents). Specifically, on the onset of symptoms in the first patient of a family, it is possible that other members give much of their support to this patient, making it more difficult to expend enough resources on those who are diagnosed later. On a broader level, home quarantine as a general practice during the pandemic presents unprecedented challenges for family households, due to inadequate space for social distancing, taxing family duties, limited care resources, and financial constraints (Hado & Feinberg, 2020; Mohile et al., 2020; Stokes & Patterson, 2020; Usher et al., 2020). These challenges are compounded if multiple family members have recognizable symptoms.

### Stress and Strain

A closely related mechanism is the stress process triggered by a family member or close tie’s illness. Research shows that stress is one of the strongest determinants of mental and physical health (Thoits, 2011). Psychological research has long identified stress as a major precedent of depression and suicidal attempting, and studies using state-of-the-art research design have consistently shown a causal relationship between prior life stressors and major depressive episodes (Hammen, 2005). Additionally, research in neuroimmunology has documented a clear link between stress and the immune systems. Stressors lasting only for minutes may trigger reactions in specific functions of the immune systems, whereas major stress-inducing life events (e.g., bereavement, cancer) can seriously disrupt individuals’ immune responses (Reiche et al., 2004; Segerstrom & Miller, 2004).

Equally importantly, a growing literature in sociology shows that social connectedness is an essential buffer against the detrimental consequences of life stressors (Thoits, 2011). Social isolation leads to perceived loneliness and stress, which may in turn put an individual at greater risk of mortality, morbidities, compromised immune functions, and cognitive decline (Barnes et al., 2004; Courtin & Knapp, 2017; House, 2001; Pressman et al., 2005; Reiche et al., 2004; Seeman, 2000; Uchino et al., 2001). By contrast, feeling connected to others is associated with stronger abilities to develop effective coping strategies, higher self-esteem, and a better sense of control, all of which are key to managing stressful situations and negative emotions (Baqutayan, 2015; Benner & Wrubel, 1989; Lazarus & DeLongis, 1983; Matud, 2004; Warren-Findlow & Issel, 2010).

In the context of COVID-19, the co-occurrence of one’s own illness alongside the illness of a loved one can exacerbate stress, which can have numerous downstream consequences. For one, stress can lead to a compromised immune system, which is a proven significant risk factor of severe COVID-19 (Esai Selvan, 2020). In addition, while social connectedness is a key mechanism that buffers individuals from stress and attendant physical morbidities, the illness of a close tie or family member can reduce a person’s perceived connectedness to others due to their own or the contact’s isolation for treatment. For instance, one study found that family members who are both diagnosed with COVID-19 but are quarantined at different places tend to report higher levels of isolation, anxiety and stress (Qiu et al., 2020).

Apart from stress induced by infection, family caregivers’ role strain in the face of intrafamilial infection of COVID-19 can incur particular health risks on both the caregivers themselves and their loved ones (Henning-Smith, 2020). As people stay home to slow the spread of COVID-19, family caregivers face added liabilities and risks. Routine tasks—such as grocery shopping, running errands, and accessing health care—now all come with new risks of infection (Q. Chen et al., 2020; Qian et al., 2020). Studies have already shown that during the early stages of the COVID-19 pandemic, caregivers in a family often experience negative emotions such as fatigue, discomfort, and helplessness (Q. Chen et al., 2020; Sun et al., 2020). These aspects of caregiver strain may spread through personal networks, negatively affecting the health of all related members (Dunkin & Anderson-Hanley, 1998). It is possible that the damaging effects of stress on the caregiver become multiplicatively greater as multiple members in the household fall ill.

### Social Exposure and Viral Load

A final mechanism through which intrafamilial infection may affect illness severity is SARS-CoV-2 viral load, which refers to the amount of virus in a person’s system. There are several reasons to expect a relationship between the infection of a family member or close tie and illness severity. Individuals who have family members or other close ties who are infected with the virus may encounter higher viral loads because they have more frequent and sustained interactions with infected people. This is likely to occur not only because of the greater likelihood of repeated and sustained exposure to infected contacts (e.g., via extended conversations) but also because of the more intimate nature of those exposures which can involve substantial exposure to respiratory droplets (e.g., via hugging, kissing, laughing, and yelling). Moreover, close contacts likely have multiple exposures to each other’s illness, including the direct exposures just described but also indirect exposure via contact with multiple contaminated surfaces (e.g., doorknobs, countertops, and other commonly shared household items).

Medical research has shown that patients’ viral loads tend to peak at the start of infection (Liu et al., 2020). In this respect, family members or close ties who contract the virus from the initial patient may have high viral loads, given that interactions are the least likely to be avoided when the latter is still in the incubation or presymptomatic period. However, it is important to note that scientists have not yet reached consensus on the impact of viral load on COVID-19 illness severity. Some studies find a positive relationship (Liu et al., 2020; Pujadas et al., 2020), while others have found little or no association (Cereda et al., 2020; He et al., 2020) or even an inverse association (Argyropoulos et al., 2020).

## Hypotheses

Applying the above insights to the context of COVID-19 patients, we expect that compared with isolates (i.e., patients who have no ties to other patients) whose loved ones remain healthy, those whose family members or other close ties are also sick with COVID-19 are subject to multiple layers of extra burden and exposure risks, including the loss of emotional and instrumental support, stress and strain, and greater viral load. We therefore propose our main hypothesis:

**Main Hypothesis:** COVID-19 patients who have more family members or other close social ties who have previously been diagnosed with COVID-19 tend to subsequently develop more severe symptoms.

We also consider a complementary hypothesis, taking into account the unique circumstances of COVID-19 as a public health emergency actively monitored by contact tracing and isolation interventions. Close contacts of a confirmed case may be more alert to their own symptoms, and in many cases are outright mandated quarantine and testing by health authorities. Moreover, if—as in our main hypothesis—individuals with infected close ties tend to experience worse symptoms, these individuals may be more likely to seek medical treatment in the earlier stages of their disease. Given that earlier diagnosis of the infection can help alleviate the accelerated progression of COVID-19 symptoms (Bi et al., 2020), we also have a complementary hypothesis:

**Complementary Hypothesis:** The diagnosis of a close tie can lead to the earlier identification and treatment of one’s own infection, which in turn protects against developing more severe symptoms.

## Method

### Data

To test these hypotheses, we use public patient-level data from the Chinese city of Shenzhen. In response to the COVID-19 pandemic, the city government has released data on individual cases on its website (https://opendata.sz.gov.cn/). These anonymized data are collected by the Shenzhen Municipal Health Commission and are made publicly available. They contain documentation of each patient’s recent travel and contact history, the timing and clinical trajectory of their illness, as well as several sociodemographic variables.

**Figure 1.**
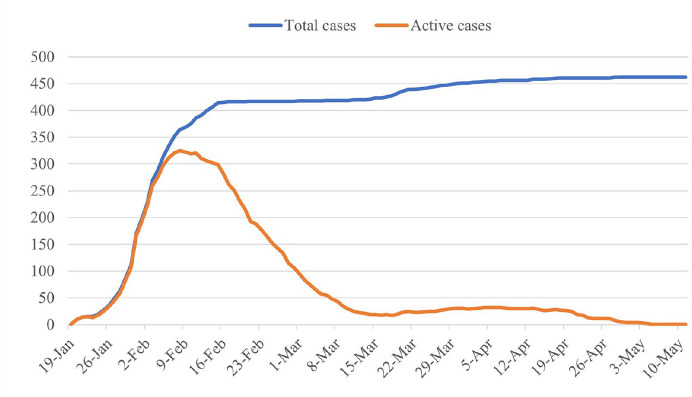
Number of COVID-19 cases in Shenzhen, China *Note*. Data are extracted directly from the summary statistics provided by the Shenzhen government website. Timing in the figure represents the date of data release and can be slightly delayed than a patient’s actual date of diagnosis.

The data cover the period between the first known case (diagnosed on January 9, 2020) to February 25, totaling 417 COVID-19 patients. February 25 is the end point because the Shenzhen government stopped releasing data on individual patients’ discharge dates from February 26 onward. While truncated, our sample captures key patterns of contact-based spread in Shenzhen for the relevant period. By late February, the spread of the virus in Shenzhen had far passed its peak (see Figure 1). Indeed, all Shenzhen cases after February 25 came from abroad and were quarantined at airport entry points, involving no reported domestic transmission. All Shenzhen patients were treated at the Third People’s Hospital of Shenzhen, and thus we do not consider variations in hospital characteristics in our analysis.

#### Patient Social Network Data

Ties between confirmed cases constitute the essential information we need to construct patient networks. On diagnosis, a Shenzhen patient reports whether they have recently been in close contact with any other known case(s), and their type of relationship to them. In our analysis, we define two patients as mutually linked whenever one patient identifies the other as a close contact, regardless of who reported whom. A key consideration here is that our patient network represents *not* so much the epidemiological chain of infection—or who infected who, as the social ties of the infected—or the prior existence of close relationships between confirmed cases. Links are recorded not just when a later case recalls contact with an earlier one but also when tied individuals test positive together. Moreover, while contact with earlier cases was the source of infection for some, many tied patients also experienced the confounding source of shared environmental exposure (e.g., a family trip to Wuhan). Furthermore, it could be the case that actual transmissions occurred between unlinked patients but escaped people’s attention precisely due to the lack of an existing relationship. We emphasize that rather than undermine our study, ignoring the directional aspect of patient networks serves our research goal of surveying the health impact of falling ill *together* with a close tie.

### Variables

#### Dependent Variables

Our main outcome of interest is patients’ illness severity. To operationalize the complementary mechanisms in our hypotheses, we derive two alternative measures for this outcome: *length of illness* (LI) versus *length of hospital stay* (LOS). The former represents the total duration from a patient’s symptom onset to their hospital discharge, whereas the latter is a patient’s number of days in hospital. For both measures, patients who remained in hospital by the end of our study period are assigned the duration of February 25 minus their corresponding starting date, and we use a time-to-event framework to account for their right censoring. Note that we also have data on whether the patient was classified as severe or critical by the Shenzhen health authorities, and results using this dichotomous measure are consistent with our LOS-based findings (see the section on sensitivity analysis). We focus on the duration measures here due to concerns about the statistical power of the small number (12 cases) of severe (8 cases) or critical (4 cases) cases.

To directly test whether the diagnosis of family members or close ties speeds up the identification of one’s own infection, we also run models predicting *time to diagnosis* (TTD). We calculate TTD as the duration between symptom onset and diagnosis. Importantly, immediate hospitalization is enforced for all confirmed cases in Shenzhen regardless of symptom severity. Hence diagnosis is equivalent to hospital admission, and the LI by definition equals the sum of LOS and TTD.

#### Social Network Predictors

While we treat our patient network as undirected, we do consider the temporal evolution of patients’ ties to avoid reverse causation. Specifically, we allow our two main network-related predictors to vary by time. For each day since a patient first showed symptoms or tested positive (corresponding respectively to LI/TTD and LOS), the variable *number of close contacts* represent how many of their close contacts were hospitalized on that particular day. Our theoretical intuition here is that individuals who have more infected contacts can be more strongly subject to the hypothesized patient network influences. We also include a variable called *number of core family members*, which counts the daily sum of parental child and spousal relationships in which a patient is involved. We specify this variable because on the one hand, the literature highlights the particularly influential role of close family ties (Connidis, 2014; Kazianga & Wahhaj, 2017; Umberson & Thomeer, 2020), and on the other hand, these are two dominant types of ties in our data.

**Table 1.** Descriptive Statistics (*N* = 417).

Variable	Description	Range	Mean/proportion	*SD*
*Dependent variables*				
Time to diagnosis (TTD)	Number of days between symptom onset and diagnosis	0-20	3.79	3.72
Length of hospital stay (LOS)	Number of days in hospital	5-47	19.93	6.39
Length of illness (LI)	Number of days between a patient’s symptom onset and their hospital discharge (LI = TTD + LOS)	10-53	23.73	7.12
*Time-varying variables*				
Infected close contacts for LI	Number of close contacts diagnosed with COVID-19 between a patient’s symptom onset and hospital discharge, by day	0-5	0.50	0.71
Infected core family member for LI	Total number of parents, children or spouse diagnosed with COVID-19 between patient’s symptom onset and hospital discharge, by day	0-2	0.39	0.55
Infected close contacts for LOS	Number of close contacts diagnosed with COVID-19 between a patient’s hospital admission and discharge, by day	0-5	0.57	0.74
Infected core family member for LOS	Total number of parents, children or spouse diagnosed with COVID-19 between a patient’s hospital admission and discharge, by day	0-2	0.45	0.57
Infected close contacts for TTD	Number of close contacts diagnosed with COVID-19 by day between a patient’s symptom onset and diagnosis	0-3	0.19	0.40
Infected core family member for TTD	Total number of parents, children or spouse diagnosed with COVID-19 between a patient’s symptom onset and diagnosis	0-2	0.14	0.35
*Time-invariant variables*				
Male	Patient’s sex on records (Male = 1; Female = 0)	0 or 1	47.48%	0.50
Age (divided by 10)	Patent’s age on records; divided by 10 for easier interpretation	1-86	4.54	1.77
*Source of virus exposure*				
	Contact with local case		3.36%	
	Contact with Wuhan case		7.43%	
	Traveled to Hubei		73.62%	
	Other travel related contact		4.08%	
	Unknown (possibly local)		11.51%	
Total cases on the first day of hospitalization (divided by 100)	Number of confirmed cases in Shenzhen on the first day of a patient’s hospitalization; divided by 10 for easier interpretation	0-336	186.32	107.76
Total cased on the first day of symptom onset (divided by 100)	Number of confirmed cases in Shenzhen on the first day of a patient’s symptom onset	0-336	124.11	107.26

*Note*. Summaries for dependent and time-invariant variables are obtained in wide form with one observation per subject. Summaries for time-varying variables are obtained in long form with multiple observations per subject and are corrected for clustering by fitting patient ID’s as random effects.

#### Control Variables

We control for several demographic and epidemiological variables potentially associated with illness severity. We include age, gender, and a five-category factor indicating a patient’s source of virus exposure. We also calculate the total number of currently hospitalized patients in Shenzhen when a patient first showed symptoms or tested positive (respectively for LI and LOS), given the possibility that the city-level context or stage of the epidemic might impact the progress of patients’ treatment or diagnosis. We divide age and total Shenzhen case count respectively by 10 and 100 when fitting our models for more meaningful interpretations.

#### Missing Values and Censoring

The data have no missing values for any predictor or outcome variables except TTD. Two out of the 417 patients were excluded for TTD analysis because they showed symptoms *after* diagnosis (through contact tracing). For LI and LOS, 262 of the 417 patients were discharged by the end of the study period. For TTD, all 415 patients who had a valid TTD measure received a diagnosis. No patient left the study during the observation period.

### Analytic Strategy

We use survival modeling for our statistical analysis because our outcomes are duration variables containing information on both the occurrence and the timing of events (see Allison, 2014, for more descriptions of the method). For multivariate modeling, we report results from the Cox proportional hazard model, a well-recognized method in survival analysis. The Cox model predicts the “hazard” of the subject experiencing an event (i.e., the probability of experiencing an event at an instantaneous time point, conditional on the subject’s not having yet experienced the event). Here, our events of interest are hospital discharge or receiving a diagnosis, and greater hazards in the estimated models correspond to *shorter* durations leading up to the event. A hazard ratio of larger than 1 indicates that a predictor in question *increases* the hazard or rate of the event occurring, or equivalently, that it *decreases* the duration needed for the event to occur; alternatively, a hazard ratio of 1 means no effect.

For bivariate analysis, we conduct log-rank tests, univariate Cox regression as well as Kaplan–Meier curve analysis. Log-rank test and Kaplan–Meier curve are common nonparametric survival analysis techniques for examining observed differences in hazards between groups, and we dichotomize our network predictors (i.e., 0 vs. 1+ contacts) for these analyses to allow for cross-group comparisons. To incorporate time-varying covariates, we prepare our data in long form for analysis of multiple observations per subject. For more accuracy in estimation, we adopt the exact partial-likelihood method to handle “tied events” (i.e., cases where two or more subjects experienced the event at the same recorded time).

We begin by visualizing the whole patient network of Shenzhen, describing basic configurations of between-patient ties and their possible relationship with patients’ LOS. We then move on to a formal test of our hypotheses using bivariate and multivariate survival models. We compare models predicting LOS and TTD to directly test the hypothesized differential role of intrafamilial connections for illness severity. Additionally, we run models predicting LI as further evidence for the copresence of these complementary mechanisms. Stata and Python were used for data preparation, R for network visualization and Stata for statistical analyses.

## Results

**Figure 2.**
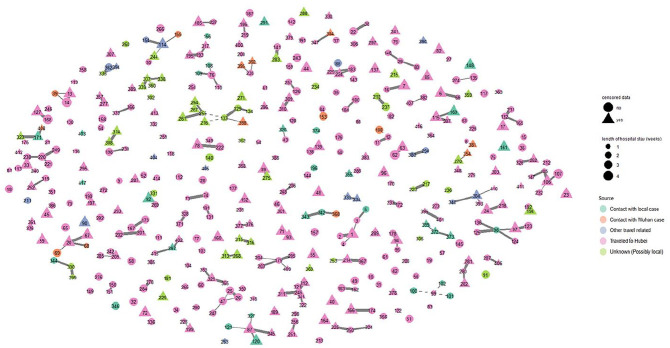
Patient network visualization of 417 cases diagnosed with COVID-19 between January 8th and February 25th in Shenzhen. *Note*. Nodes represent confirmed COVID-19 cases in Shenzhen, and edges represent social ties between cases reported by patients. We manipulate the size, shape and color of the nodes and edges to depict the distribution of the source of virus exposure, relationship type, length of hospital stay (LOS), and whether data were right censored (i.e., patient still in hospital by the end of February 25th). Types of relationship, from the thickest to dotted lines, are: spousal, parent–child, other-kin, and nonkin. The number labels for nodes denote the sequence of each case’s diagnosis.

We visualize the city-level network of Shenzhen COVID-19 patients in Figure 2. Nodes in the graph represent patients whereas edges represent between-patient links. Here, we number our nodes to refer to the sequential order of patients’ diagnosis and link two patients as long as one of them ever identified the other as a close contact. We manipulate the size, shape, and color of the nodes and edges to depict the distribution of several relevant variables, including the source of virus exposure, relationship type, LOS, and “censored data.” The “censored data” measure is added specifically for this graph to distinguish whether the patient was still hospitalized as of February 25th, the end of our observation period.

Several patterns are immediately visible. First, the family indeed appears to be a predominant locus of transmission. Among the 56% of patients who are nonisolates, the type of relationship that links them is almost exclusively kin ties (95%), with spousal ties (36%) and child–parent ties (39%) being particularly salient. Second, community and local spread remains relatively limited in Shenzhen. A significant proportion of nodes are isolates (44%) or dyads (24%), and a history of travel to Hubei proves the absolute top source of exposure for individuals (74%). Third, triangle nodes tend to be larger than circle ones, meaning that patients who were yet to be discharged by February 25 also have longer LOS (25.38 vs. 18.89 days, *p* < .001). This highlights the necessity to adopt a survival framework in statistical analyses to correct for right censoring.

### Bivariate Analysis

Results from bivariate analysis corroborate both our main and complementary hypotheses. Across the log-rank tests and univariate Cox models, Table 2 reveals significantly higher rates of hospital discharge for LOS among patients with no infected contacts relative to those with at least one infected close contact or core family member (*p* < .05). By contrast, the hazard of getting a COVID-19 diagnosis since one’s symptom onset (i.e., TTD) proves much higher for patients with at least one close contact or core family member who was also diagnosed (*p* < .001). For example, the univariate Cox estimates for TTD in Table 2 indicate that patients whose close contacts or core family members tested positive were diagnosed about three times (*b* = 2.91, *p* < .001 or *b* = 2.93, *p* < .001) as fast as patients without infected close contacts.

**Table 2.** Bivariate Tests for Equality of Survival Curves by Network Features.

Outcomes	Log-rank test			Univariate Cox model	
	No infected close contacts		1 + Infected close contacts	Number of infected close contacts	
	Median survival time		*p*	Hazard ratio	*p*
Length of illness (LI)	26	28	.117	0.83	.053
Length of hospital stay (LOS)	21	24	.019	0.78	.0090
Time to diagnosis (TTD)	5	1	<.0001	2.91	<.0001
Outcomes	Log-rank test			Univariate Cox model	
	No infected core family members		1 + Infected core family members	Number of infected core family members	
	Median survival time		*p*	Hazard ratio	*p*
Length of illness (LI)	26	29	.052	0.82	.099
Length of hospital stay (LOS)	21	24	.0060	0.75	.014
Time to diagnosis (TTD)	5	1	<.0001	2.93	<.0001

*Note*. This table compares the illness severity (length of hospital stay, the length of illness) and diagnosis timing (time to diagnosis) by whether a patient has at least one close tie or core family member who is diagnosed with COVID-19. According to both the Log-rank tests and the univariate Cox model, patients with at least one infected close contact or core family member have significantly longer length of hospital stay but significantly shorter time to diagnosis. There is some weak evidence that these patients also have longer illness duration, but the difference is not statistically significant at a 0.05 level. All 417 patients were included in the analysis for LI and LOS, out of whom 262 experienced the event of hospital discharge. 415 patients were included in the analysis for TTD, all of whom experienced the event of receiving a COVID-19 diagnosis. No patient left the study during the observation period.

**Figure 3.**
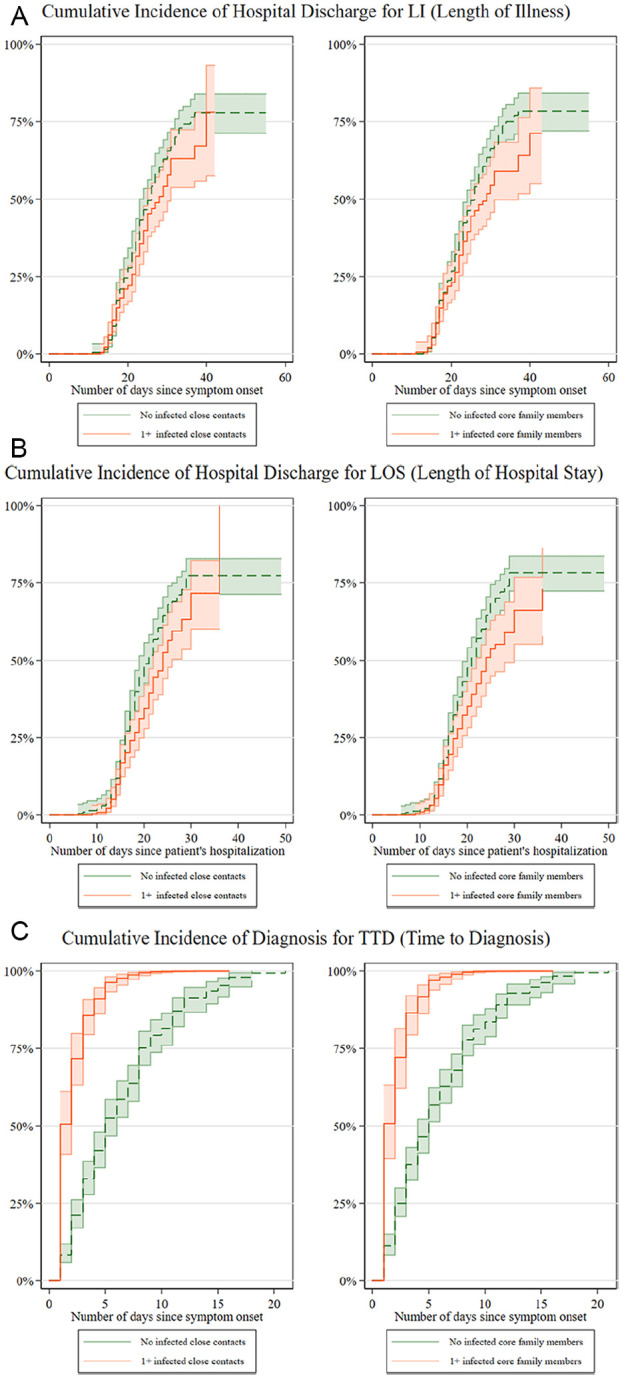
Kaplan–Meier failure curve for and LI (length of illness), LOS (length of hospital stay), and TTD (time to diagnosis) by network features. *Note*. These panels show observed cumulative incidences of hospital discharge (for length of illness and length of hospital stay) and diagnosis (for time to diagnosis) between patients with versus without at least one infected close contact or core family member. Curves are shown with standard errors and 95% confidence intervals. 417 patients were included in the analysis for LI and LOS, out of whom 262 experienced the event of hospital discharge. 415 patients were included in the analysis for TTD, all of whom experienced the event of receiving a COVID-19 diagnosis. No patient left the study during the observation period.

These patterns are also well illustrated in the Kaplan–Meier failure curves in Figure 3. In the LOS panels, the solid curves are consistently taller than the dotted curves, indicating a higher probability of hospital discharge for those with no infected close contacts or core family members since initial symptom onset or hospital admission. Meanwhile, a still larger gap between the curves—albeit in the opposite direction—characterizes the TTD panels.

Results regarding LI are less straightforward. While similar patterns of lower rates of hospital discharge are observed for the LI panels in Figure 3, bivariate tests in Table 2 show less significant (*p* < .10 or *p* = .117) differences in terms of illness duration between nonconnected patients and those who have infected contacts. These results are not surprising, given that LI can be viewed as a combination of the opposing dynamics exhibited in the LOS and TTD processes.

### Multivariate Analysis

In Table 3, we contrast Cox regression models for LOS (Models 1 and 2) against those predicting TTD (Models 3 and 4). Once again confirming our main hypothesis that patients with infected contacts are more likely to develop more severe symptoms, Models 1 and 2 suggest a significant (*p* < .05) and negative association between the rate of hospital discharge and the infections of their close contacts or close family members. According to Model 1, net of age, gender, source of virus exposure, and the total number of city-level cases at the time of one’s symptom onset, having one additional close contact who is also infected with the virus decreases a patient’s hazard of hospital discharge by 22% (= 1 − .78). The estimated drop in hazard is slightly larger at 25% (= 1 − .75) if the infected contact is a spouse, parent, or child (Model 2). In both cases, the network effect is rather strong, comparable to the risk of extended LOS induced by being 10 years senior in age. Also, age proves the only control variable that is significant in Models 1 and 2: Every 10 years increase in age reduces a patient’s rate of hospital discharge by 20% (= 1 − .80, *p* < .001).

**Table 3.** Hazard Ratios for Multivariate Cox Models on LOS and on TTD.

	Models for LOS		Models for TTD	
	Model 1	Model 2	Model 3	Model 4
Infected close contacts	0.78* [0.64, 0.94]		5.19*** [3.94, 6.85]	
Infected core family members		0.75* [0.59, 0.96]		4.36*** [3.25,5.84]
Age (divided by 10)	0.80*** [0.74, 0.86]	0.80*** [0.75, 0.87]	1.00 [0.93, 1.08]	1.00 [0.93, 1.08]
Male	1.06 [0.82, 1.38]	1.07 [0.83, 1.38]	1.05 [0.83, 1.34]	1.04 [0.82, 1.32]
Total cases on the first day of hospitalization (divided by 100)	0.93 [0.81, 1.06]	0.95 [0.83, 1.09]		
Total cases on the first day of symptoms onset (divided by 100)			1.32*** [1.16, 1.51]	1.41*** [1.24, 1.60]
Source of exposure (*Ref = Contact with local case)*				
Contact with Wuhan case	0.96 [0.38, 2.44]	1.03 [0.40, 2.60]	2.70* [1.21, 6.02]	1.85 [0.82, 4.19]
Traveled to Hubei	1.22 [0.56, 2.67]	1.33 [0.60, 2.91]	3.06** [1.52, 6.16]	1.85^†^ [0.90, 3.79]
Other travel related	0.94 [0.33, 2.70]	0.96 [0.33, 2.77]	2.15^†^ [0.89, 5.24]	1.20 [0.49, 2.95]
Unknown (Possibly local)	0.87 [0.36, 2.10]	0.93 [0.39, 2.25]	1.78 [0.84, 3.78]	1.18 [0.55, 2.56]
Observations	8,884	8,884	1,999	1,999
Subjects	417	417	415	415
Failure	262	262	415	415
AIC	1841.6	1843.6	1726.1	1771.5
BIC	1898.3	1900.3	1770.9	1816.3

*Note*. This table uses multivariate Cox analyses to investigate whether the number of infected close ties or core family members is associated with patients’ length of hospital stay (LOS) and time to diagnosis (TTD). We juxtapose results for these two outcomes because of our hypotheses that illness severity is exacerbated by the infection of family members or close ties, but the progression of the disease can be alleviated by the quickened diagnosis among those with familial or close ties to other patients. The models in Table 2 provide evidence for these hypotheses, after controlling for possible confounders. The hazard of hospital discharge significantly decreases, with the presence of infected close contacts or infected core family members, in contrast to the significantly increased rates of diagnosis. In particular, Models 1 and 2 estimate that the extension in hospital stay due to intrafamilial or close tie infection is comparable to, and indeed slightly larger than that caused by a 10-year increase in age. Exponentiated coefficients; 95% confidence intervals in brackets; All 417 patients are at risk of hospital discharge for LOS, and 415 patients are at risk of receiving a diagnosis for TTD. Data are prepared in long form with multiple observations per subject. Tied events are handled with the exact partial-likelihood method to increase estimation accuracy. AIC = Akaike information criterion; BIC = Bayes Information criterion.

† *p* < .10. ^*^p < .05. ^**^p < .01. ^***^p < .001.

On the other hand, Models 3 and 4 provide support for our complementary analysis regarding the faster diagnosis process for patients with infected ties. These models show significantly enhanced rates of diagnosis for patients with close ties to other patients (*p* < .001). Controlling for other variables, each additional close contact (Model 3) or core family member (Model 4) who is also diagnosed with COVID-19 multiplies one’s rate of receiving a diagnosis by 5.19 and 4.36, respectively. Additionally, the source of virus exposure (Model 3) and the number of city-level active cases (Models 3 and 4) also emerge as significant predictors of the rate of diagnosis. Compared with patients whose source of exposure was contact with local Shenzhen cases, Model 3 shows that patients who had a history of travel to Hubei (*p* < .01, confidence interval [CI: 1.52, 6.16]) and those who had prior contact with Wuhan cases (*p* < .05, CI [1.21, 6.02]) have significantly higher rates of being diagnosed. Regarding city-level active cases, Models 3 and 4 predict a net increase in the rate of diagnosis by, respectively, 32% and 41% with every 100 additional hospitalized cases in Shenzhen.

In Table 4, we test a set of models for LI to further explore how the two hypothesized processes may simultaneously operate to impact patients’ illness severity. Juxtaposing Models 5 and 7, respectively, against Models 6 and 8, the coefficients for network predictors are consistently significant in Models 6 and 8 where TTD is included as a control variable (*p* < .05). On the other hand, without TTD as a control, Model 5 (*p* = .099) and Model 7 (*p* = .113) show no relationship at the 0.05 level between the rate of recovery and the number of infected close contacts or core family members. All four models suggest a prolonged LI if a patient has more close contacts or core family members who are also diagnosed with COVID-19, but the effect sizes are larger after TTD is controlled for. For example, Model 8 predicts a 23% (= 1 − 0.77) decrease in the rate of recovery with each additional core family member being infected, whereas the corresponding figure is 18% according to Model 7.

**Table 4.** Hazard Ratios for Multivariate Cox Models on LI (Length of Illness).

	Model 5	Model 6	Model 7	Model 8
Infected close contacts	0.85^†^ [0.70, 1.03]	0.81* [0.67, 0.98]		
Infected core family members			0.82 [0.64, 1.05]	0.77* [0.61, 0.98]
Time to diagnosis		0.90*** [0.87, 0.94]		0.90*** [0.87, 0.94]
Age (divided by 10)	0.81*** [0.75, 0.87]	0.79*** [0.73, 0.86]	0.81*** [0.75, 0.87]	0.80*** [0.74, 0.86]
Male	1.01 [0.79, 1.31]	1.04 [0.80, 1.34]	1.01 [0.79, 1.31]	1.04 [0.81, 1.34]
Total cases on the first day of symptoms onset (divided by 100)	1.15^†^ [0.99, 1.33]	1.01 [0.86, 1.18]	1.16 [1.00, 1.35]	1.03 [0.88, 1.21]
Source of exposure (*Ref = Contact with local case)*				
Contact with Wuhan case	0.93 [0.37, 2.36]	0.95 [0.37, 2.42]	0.97 [0.38, 2.46]	1.01 [0.40, 2.57]
Traveled to Hubei	1.22 [0.55, 2.69]	1.21 [0.55, 2.68]	1.28 [0.58, 2.84]	1.31 [0.59, 2.90]
Other travel related	0.81 [0.28, 2.34]	1.00 [0.34, 2.90]	0.82 [0.28, 2.39]	1.04 [0.36, 3.05]
Unknown (possibly local)	0.71 [0.29, 1.71]	0.87 [0.36, 2.11]	0.74 [0.31, 1.79]	0.92 [0.38, 2.25]
Observations	10,466	10,466	10,466	10,466
Subjects	417	417	417	417
Failure (hospital discharge)	262	262	262	262
AIC	1891.5	1863.3	1891.9	1863.8
BIC	1949.6	1928.6	1949.9	1929.1

*Note*. This table uses multivariate Cox analyses to investigate whether the number of infected close ties or core family members affects total illness duration, with (Models 6 and 8) versus without (Models 5 and 7) time to diagnosis (TTD) as a control variable. TTD is singled out because of the hypothesis that the intrinsically lengthened illness severity as a result of a family member or close tie’s infection can be remedied by the shortened time to diagnosis among those involved in intrafamilial or closely tied infection. The models suggest evidence for this hypothesis. Models 6 and 8 show a significant and negative effect of family members’ or close ties’ infection on patient’s hazard of hospital discharge, which is absent in Models 5 and 7. Exponentiated coefficients; 95% confidence intervals in brackets; All 417 patients are at risk of hospital discharge for the outcome variable. Data are prepared in long form with multiple observations per subject. Tied events are handled with the exact partial-likelihood method to increase estimation accuracy. AIC = Akaike information criterion; BIC = Bayes Information criterion.

† *p* < .10. ^*^p < .05. ^**^p < .01. ^***^p < .001.

### Sensitivity Analysis

An important concern regarding our analysis is that LOS might not be a valid measure of illness severity. One may suspect that early diagnosis artificially inflates LOS regardless of illness severity, given that the Shenzhen authorities hospitalize all patients who test positive. In particular, in the absence of effective antiviral treatment capable of shortening the duration of COVID-19, early diagnosis could be a chief factor accounting for longer LOS.

We conduct two sensitivity analyses in response to this concern (Table 5). First, we rerun LOS-related Cox models, adding TTD as a control variable (Models S1 and S2). Second, we run a logistic regression predicting whether a case is marked as severe or critical during their hospitalization (Models S3 and S4), using the Firth algorithm to account for the rarity of these cases(Wang, 2014). In both analyses, we again observe a positive association between severity and close ties’ infections. Models S1 and S2 for LOS show delayed hospital discharge for higher counts of infected close contacts (*p* < .05, CI [.64, .94]) or core family members (*p* < .05, CI [.59, .97]), with no effect of TTD on LOS (*p* = .80 for Model S1, *p* = .84 for Model S2). Similarly, logistic regressions in Models S3 and S4 indicate that the odds of being a severe or critical case multiply by 1.92 or 2.5 with each additional infected close contact (*p* < .05) or core family members (*p* < .10), but are unaffected by the rate of diagnosis (*p* = .86 for Model S3, *p* = 0.71 for Model S4). Additionally, the logit models identify city-level case count at the time of a patient’s diagnosis as a negative predictor of case severity (*p* < .01).

**Table 5.** Sensitivity Analyses for Illness Severity Findings Based on LOS (Length of Hospital).

	Hazard ratios for Cox model on LOS (with TTD as a control)		Odds ratios for Logistic model on severe or critical cases	
	Model S1	Model S2	Model S3	Model S4
Infected close contacts	0.78* [0.64, 0.94]		1.92* [1.04, 3.54]	
Infected core family members		0.76* [0.59, 0.97]		2.50^†^ [0.96,6.47]
Time to diagnosis	1.00 [0.97, 1.04]	1.00 [0.97, 1.04]	1.02 [0.81, 1.28]	1.04 [0.84, 1.30]
Age (divided by 10)	0.80*** [0.74, 0.86]	0.80*** [0.74, 0.87]	2.65** [1.28, 5.49]	2.44* [1.21, 4.94]
Male	1.06 [0.82, 1.37]	1.07 [0.83, 1.38]	1.75 [0.35, 8.73]	2.31 [0.48, 11.0]
Total cases on first day of hospitalization (divided by 100)	0.92 [0.80, 1.06]	0.94 [0.82, 1.09]	0.088** [0.017, 0.45]	0.074** [0.014, 0.41]
Source of exposure (*Ref = Contact with local case)*				
Contact with Wuhan case	0.96 [0.38, 2.43]	1.02 [0.40, 2.59]	2.59 [0.073, 92.5]	1.83 [0.053, 62.8]
Traveled to Hubei	1.21 [0.55, 2.66]	1.31 [0.60, 2.90]	0.071 [0.0019, 2.69]	0.041 [0.0011, 1.51]
Other travel related	0.92 [0.31, 2.68]	0.94 [0.32, 2.76]	0.79 [0.011, 58.4]	0.37 [0.0064, 21.5]
Unknown (possibly local)	0.86 [0.35, 2.09]	0.92 [0.38, 2.24]	4.00 [0.10, 158.3]	3.54 [0.11, 118.7]
Observations	8,884	8,884	417	417
Subjects	417	417	417	117
Failure	262	262	—	—
AIC	1843.5	1845.5	61.0	62.7
BIC	1907.3	1909.3	101.3	103.0

*Note*. Exponentiated coefficients; 95% confidence intervals in brackets; All 417 patients are at risk of hospital discharge for LOS in the Cox models. All Firth logit models to modify the biases due to small number (12) of severe or critical cases. AIC = Akaike information criterion; BIC = Bayes Information criterion.

† *p* < .10. ^*^p < .05. ^**^p < .01. ^***^p < .001.

**Figure 4.**
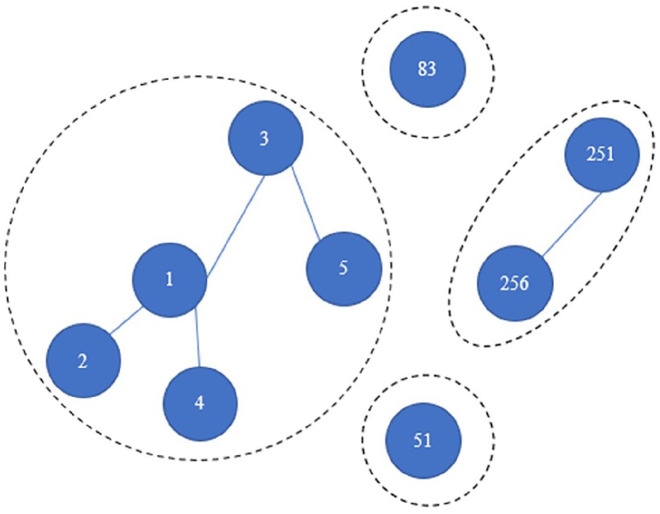
Illustration for locally connected networks. *Note*. This is a subgraph of the Shenzhen patient network visualization in the main text. Here, there are four locally connected networks (“groups”), two of which involve isolates. Each group ID’s will be a random effect predictor in our sensitivity test of the Cox frailty models.

Another key concern relates to the potential problem of clustering in our data, the presence of which would violate the independence assumption of conventional Cox models. Specifically, patients who belong to the same locally connected network (“group”) may display more similar health behavior than randomly selected patients (Figure 4). To account for this possibility, we run null Cox shared frailty models for all three outcomes, fitting group ID as random-effect dummy indicators. In no cases do we detect evidence of clustering (*p* > 0.4). The conventional Cox models presented in our analyses are therefore adequate for our purposes.

Finally, we also tested whether the omission of asymptotic cases or the use of dummy measures of network predictors (i.e., 0 vs. 1+ close contacts or core family members) alters our main results. For both of these concerns, we reran all our Cox models, and we note that our main findings prove robust to these sensitivity checks (results available on request from the corresponding author).

## Discussion

While previous studies have emphasized the prevalence of intrafamilial transmission in the spread of COVID-19, this article has examined whether the infection of family members or close ties influences the severity of COVID-19 patients’ symptoms. This question is not only of public health urgency but also an empirical prediction derivable from theories of social networks and health. Consistent with our main hypothesis, our analyses suggest that having familial or close ties to other confirmed cases exacerbates the severity of one’s own subsequent illness, whether measured in terms of hospital stay length (LOS) as in our main results or the dichotomous measure of severe or critical symptoms as in the sensitivity analysis. At the same time, we find evidence, in line with our complementary hypothesis, that the infection of family members or close contacts also shortens one’s TTD. Combined, these two forces counteract each other, resulting in no overall significant association between patients’ total LI and between-patient ties.

These results have important implications for public health interventions. First, adding to previously reported risk factors—such as older age, being male, certain clinical conditions, obesity, and non-White ethnicity (Esai Selvan, 2020; Tian et al., 2020; Zhou et al., 2020)—we find that having close social connectedness to other patients may be another risk factor for severe COVID-19. The predictive power of this factor is visible across all Cox and logistic models, with effect sizes consistently comparable to, and oftentimes exceeding, that of being 10 years senior in age. As for clinical practice, this finding calls for the active screening of, as well as extra care to, patients who may have family members or loved ones also testing positive, as these individuals can be more vulnerable to severe symptoms.

Second, our finding about time to diagnosis echoes but also extends prior studies’ call for early detection, isolation, and contact tracing (Bi et al., 2020). The multivariate Cox models estimate that patients with hospitalized close contacts or family members were 4 to 5 times faster in identifying their own infection, which played a decisive role in remedying the intrinsically detrimental health consequence of intrafamilial or closely tied infections. Notably, our data are from Shenzhen, where quarantine and testing are imposed on all close contacts of each confirmed case. In other settings with less vigorous public health strategies in place, one might expect even more negative outcomes caused by the association of between-patient ties with COVID-19 symptom severity.

Two additional findings are worth highlighting. First, despite their being a higher-risk group for severe COVID-19, we found that older adults were not getting tested at a greater rate than younger adults. This underlines the importance of improving older adults’ testing rate, particularly given the recent finding of higher seropositivity among adults over 65 in the general population (Xu et al., 2020). Second, the number of city-level active cases at the time of a patient’s infection proved a protective factor in our data, associated with accelerated TTD and lower likelihoods of being a severe or critical case. This runs counter to the common imagery of hospital overcrowding as cases climb, suggesting instead that with strong containment measures and sufficient hospital capacity, higher case counts need not lead to worsened patient outcomes.

Our study has several limitations. First, due to data unavailability, we cannot control for underlying medical conditions, a key risk factor for severe COVID-19. Second, our data contain all diagnosed cases of Shenzhen patients over the observed period, but undetected cases possibly existed in large numbers, especially with reference to recent evidence from Wuhan, China that many infected cases were undiagnosed (Duan et al., 2021). Nonetheless, our substantive conclusions are unlikely to be refuted by the censoring of undetected cases. To the extent that contact tracing is facilitated among closely related people as opposed to among distant contacts or strangers, undetected cases are more likely to be isolates in our study (i.e., patients who do not have close ties or family members diagnosed). On the other hand, the fact that these cases remain undetected could imply that they are less severe or asymptotic to begin with. If anything, the inclusion of undiagnosed cases most likely further magnifies our current estimates.

Third, while we know that familial tie is the dominant type of relationship between tied patients, we lack information on whether patients share the same household. The diagnosis of a coresident family member likely exerts additional impact on the gravity of one’s own illness, a conjecture that we nonetheless cannot test in view of the current data. More generally, data with more detailed demographic variables such as co-residency, the type of dwelling, marital status, and socioeconomic status would allow researchers to further unveil the dynamics and consequences of intrafamilial transmission.

Finally, we model our network predictors as time-varying covariates to mitigate reverse causation, but as in any study with nonexperimental data, endogeneity can still be an issue. For example, our time-dependent modeling strategy precludes cases where one patient develops more severe symptoms before or after the hospital discharge of their diagnosed family member, but ambiguities in time sequence could remain, due to reasons like varying incubation periods between family members, as well as the fact that one can presumably get the illness twice.

Despite these limitations, this study expands our understanding of the role that social networks play in the COVID-19 patient outcomes. We have highlighted the potential role of familial and close social ties in COVID-19 severity, but a variety of other COVID-19-related questions may also benefit from this network perspective. For example, this study treated familial and close ties as an explanatory variable, but intrafamilial infection also deserves attention as an explanandum. It is worth exploring whether certain characteristics of an individual’s personal network predispose them to a higher likelihood of clustered infections, and whether such network features disproportionately appear within specific sociodemographic groups. Also, although our main finding supports the idea that close ties to other COVID-19 patients exacerbates illness severity, we cannot actually observe the underlying social mechanisms. We encourage future researchers, especially those with more fine-grained data, to explore these processes. Research effort in these directions, we believe, will shed important light on the undoubtedly numerous, but yet unknown, social determinants and consequences of COVID-19.
